# MicroRNA-452 promotes stem-like cells of hepatocellular carcinoma by inhibiting Sox7 involving Wnt/β-catenin signaling pathway

**DOI:** 10.18632/oncotarget.8584

**Published:** 2016-04-05

**Authors:** Zhiyun Zheng, Jimin Liu, Zhe Yang, Limin Wu, Haiyang Xie, Chaozhe Jiang, Binyi Lin, Tianchi Chen, Chunyang Xing, Zhikun Liu, Penghong Song, Shengyong Yin, Shusen Zheng, Lin Zhou

**Affiliations:** ^1^ Division of Hepatobiliary and Pancreatic Surgery, Department of Surgery, First Affiliated Hospital, School of Medicine, Zhejiang University, Hangzhou, China; ^2^ Department of Pathology and Molecular Medicine, Faculty of Health Science, McMaster University, Hamilton, Ontario, Canada; ^3^ Collaborative Innovation Center for Diagnosis and Treatment of Infectious Diseases, Hangzhou, China

**Keywords:** miRNAs, liver cancer, CSCs, β-catenin, ATRA

## Abstract

The decrease of microRNA-452 (miR-452) in gliomas promoted stem-like features and tumorigenesis. However, the role of miR-452, especially in regulating cancer stem cells (CSCs) in hepatocellular carcinoma (HCC) remains ambiguous. We enriched stem-like HCC cells by serial passages of hepatospheres with chemotherapeutic agents. Stem-like characteristics including the capabilities of chemo-resistance, stemness-related gene expression profiling, self-renewal, tumorigenicity and metastasis formation were detected. MiR-452 was markedly increased in the chemo-resistant hepatospheres and human HCC tissues. and the overexpression of miR-452 in HCC patients predicted poor overall survival. MiR-452 significantly promoted stem-like characteristics *in vitro* and *in vivo*. Further, *Sox7* was identified as the direct target of miR-452, which could physically bind with β-catenin and TCF4 in the nucleus and then inhibit the activity of Wnt/β-catenin signaling pathway. Finally, the combined chemotherapy of doxorubicin and all-trans retinoic acid (ATRA) showed dramatically efficiency in suppressing HCC metastasis. These data suggested that miR-452 promoted stem-like traits of HCC, which might be a potential therapeutic target for HCC. The combination of doxorubicin and ATRA might be a promising therapy in HCC management.

## INTRODUCTION

Hepatocellular carcinoma (HCC) was one of the most devastating malignancies with extremely high cancer related death worldwide [1]. Most of the burdens were clustered in parts of Asia and sub-Saharan Africa, and even half of these cases and deaths were estimated to occur in China as a result of chronically HBV infection [1]. Though, better diagnostic standard and improvements in treatment had been accomplished during the past several decades [2], high rates of recurrence and mortality were still the main hurdles for the long term survival of HCC patients [2, 3]. Recent advances speculated that tumor recurrence and metastasis might be due to the existence of cancer stem cells (CSCs) [4–7], who owned the capabilities of self-renewal, differentiation and chemo-resistance, have been identified in various malignancies [5, 8]. MicroRNAs (miRNAs) were a class of conserved, small non-coding RNAs, approximately 22 nucleotides, which could inhibit gene expression by binding in 3′ untranslated region (3′UTR) of the target mRNAs [9, 10]. Accruing evidences demonstrated that miRNAs played vital roles in regulating self-renewal and tumorigenesis of CSCs [11]. MicroRNA-452 (miR-452) was reported that its decrease enhanced the stem-like characteristics and tumorigenicity of gliomas [12]. Intriguingly, miR-452 was down-regulated in gliomas [12], breast cancer [13], while its expression increased in HCC [14], clear cell renal cell carcinoma [15], esophageal cancer [16], urothelial carcinoma [17] and prostate cancer stem cells [18]. SOX7 was identified as a key transcription factor in a variety of developmental processes, such as cardiogenesis, hematopoiesis, myogenesis, vasculogenesis, and endoderm differentiation [19]. Moreover, the frequent decline of SOX7 expression in human malignancies suggested that SOX7 might be an emerging tumor suppressor in oncogenesis [19, 20]. Wnt/β-catenin signaling was reported to exert crucial roles in the maintenance of self-renewal for stem cells and CSCs [21–23]. All-trans retinoic acid (ATRA) had got a great success in treating acute promyelocytic leukemia by inducing naive tumor cells differentiation [24], which might also be effective in the treatment of solid tumors.

The goal of our study was to explore the molecular mechanisms related to the miR-452 over-expression in regulating CSCs in HCC.

In our study, we demonstrated that miR-452 could markedly promote the stemness of HCC cells including the abilities of chemo-resistance, self-renewal and metastasis by targeting *Sox7* involving Wnt/β-catenin signaling pathway.

## RESULTS

### MiR-452 was overexpressed in HCC cells accumulated by serial passages of hepatospheres combined with chemotherapy *in vitro*

**Figure 1:**
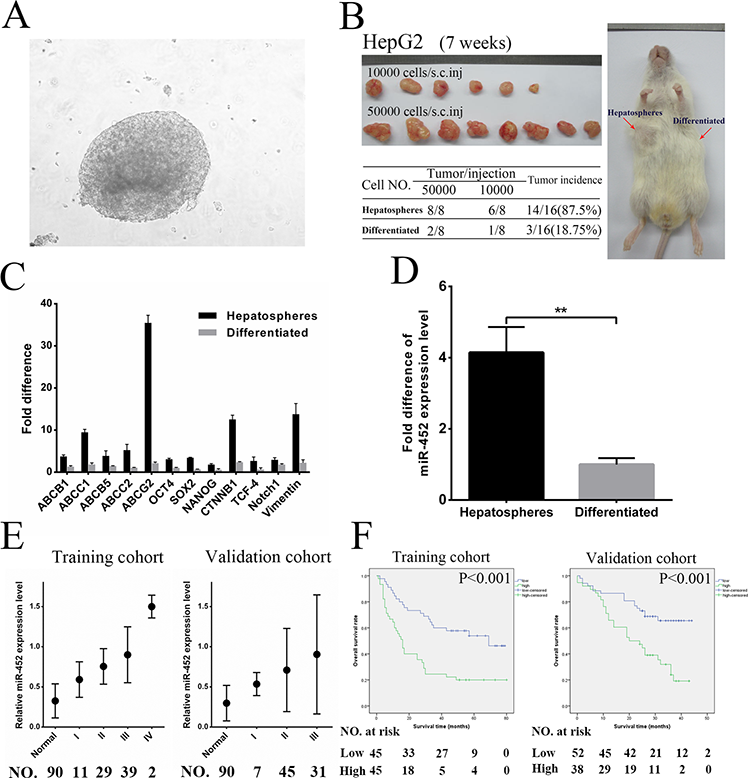
MiR-452 indicated poor survival in HCC (**A**) Hepatospheres were obtained from HepG2 cells after 20 serial passages in serum-free medium combined with doxorubicin and sorafenib. Hepatospheres with (**B**) higher capability of tumorigenicity, (**C**) higher expression of stemness-related genes, and (**D**) markedly overexpression of miR-452 compared with the differentiated clones. (**E**) Analysis of miR-452 expression in adjacent normal liver tissues and HCC specimens in accordance with different TNM stages both in the training and validation cohort. (**F**) Kaplan Meier analysis of HCC patients with high- versus low-expression of miR-452 both in training cohort (*P* < 0.001) and validation cohort (*P* < 0.001).

According to the distinct hallmarks of stem/progenitor cells such as self-renewal and chemoresistance, we enriched the liver CSCs of HepG2 upon 20 serial passages in serum-free medium combined with 2 mg/mL of doxorubicin and 5 μmol/L sorafenib (Figure 1A). Liver CSC subpopulations in the chemo-resistant hepatospheres was obtained as identified by a significantly enhanced tumorigenic ability, self-renewal, expression of stemness-related genes and liver CSC markers (Figure 1B, 1C and, Supplementary Figure S1A). Our previous data obtained by Illumina Solexa sequencing suggested that miR-452 was obviously up-regulated in HCC tissues comparing with their adjacent normal tissues (data not shown). Considering the reported biological function of miR-452, we then demonstrated that miR-452 was also markedly overexpressed in chemo-resistant hepatospheres in comparison with the differentiated HepG2 cells by qPCR (Figure 1D).

### MiR-452 up-regulation in HCC indicated poor patient prognosis

We first found that miR-452 was significantly increased in HCC cell lines comparing with that in L02 (Supplementary Figure S1B). Then, miR-452 was also overexpressed in HCC tissues than adjacent normal tissues both in training cohort and validation cohort by ISH (Supplementary Figure S1C). The representative ISH and HE graphs were presented in Supplementary Figure S1D for training cohort and Supplementary Figure S1E for validation cohort. Moreover, high miR-452 expression was positively correlated with poor patient survival (*p =* 0.002; *p =* 0.020; Table 1) and advanced TNM stages (*p =* 0.019; *p =* 0.023; Figure 1E; Supplementary Table S1) in both cohorts. Kaplan Meier curves showed that miR-452 overexpression was also associated with poor overall survival of both cohorts (*p* < 0.001; *p* < 0.001; Figure 1F).

**Table 1:** Patient characteristics with respect to overall survival

Variables	Training cohort			Validation cohort		
	Univariate^a^	Multivariate^b^		Univariate^a^	Multivariate^b^	
	*P* value	HR (95% CI)	*P* value	*P* value	HR (95% CI)	*P* value
Age, years (> 50)	0.850			0.367		
Gender (Male)	0.629			0.080		
Maximal tumor size (> 5 cm)	0.006		0.479	0.014		0.637
Tumor number (≥ 2)	0.160			0.489		
Tumor differentiation (Poor)	0.303			0.020		0.267
PVTT (Yes)	< 0.001		0.071	0.004		0.117
TNM stage (I/II/III/IV)	< 0.001	1.880 (1.177–3.003)	0.008	0.002	1.931 (1.047–3.561)	0.035
miR-452expression (low/high)	< 0.001	2.907 (1.494–5.658)	0.002	0.001	2.313 (1.144–4.676)	0.020

a univariate cox regression analysis;

b multivariate cox regression analysis;

### MiR-452 promoted stem-like characteristics of HCC cells

**Figure 2:**
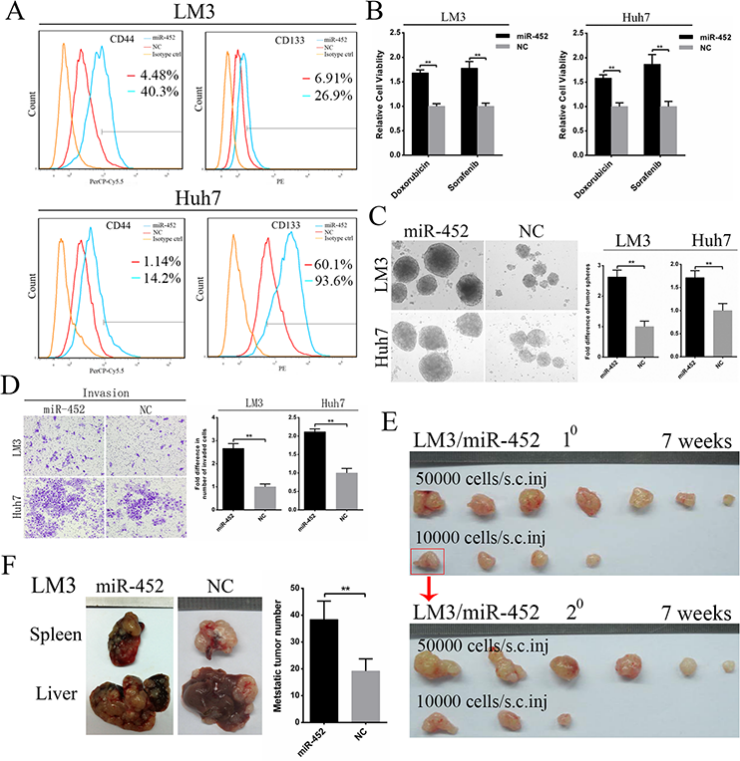
Up-regulation of miR-452 in HCC (**A**) The percentage of CD44+ and CD133+ cells markedly increased upon miR-452 up-regulation both in LM3 and Huh7. (**B**) MiR-452 overexpressed HCC cells showed higher chemoresistance to doxorubicin and sorafenib. (**C**) The self-renewal capability of LM3 and Huh7 was significantly enhanced after miR-452 expression increased in tumor sphere assay. (**D**) miR-452 efficiently promoted HCC invasion *in vitro*. (**E**) The capability of tumorigenicity significantly enhanced both in the primary and secondary xenograft model of LM3/miR-452 cells in comparison with LM3/NC cells. (**F**) *In vivo* metastasis assay, miR-452 distinctly aggravated the metastasis of LM3 cells (*n =* 5).

We established two miR-452 stable expression cell lines (HCC-LM3/miR-452, Huh7/miR-452) and their corresponding negative controls (HCC-LM3/NC, Huh7/NC) and validated the efficiency (Supplementary Figure S2A). First, FACS analysis showed that the proportion of CD44+ and CD133+ subpopulations were significantly elevated in both LM3/miR-452 and Huh7/miR-452 groups (Figure 2A), but not in CD90+ and EpCAM+ subpopulation (Supplementary Figure S2B, 2C). Then, we found that miR-452 promoted a profile of stemness-related genes expression (Supplementary Table S2). Moreover, miR-452 was also markedly increased in CD44+ and CD133+ cells than that in CD44– and CD133– cells sorted from LM3 and Huh7 by MACS (Supplementary Figure S2D). Similar results were also observed from one fresh clinical HCC sample, termed patient#1 (Supplementary Figure S2D). LM3/miR-452, Huh7/miR-452 were much more chemo-resistant than LM3/NC, Huh7/NC cells treated with either 2 mg/ml doxorubicin or 5 μM sorafenib (Figure 2B). Furthermore, tumor sphere assay showed that greater size and more hepatospheres were obtained in LM3/miR-452 and Huh7/miR-452 cells (Figure 2C). Notably, miR-452 also efficiently promoted the migration (Supplementary Figure S2E) and invasion (Figure 2D) of HCC cells *in vitro*. *In vivo* xenograft model, as few as 1000 LM3/miR-452 cells were enough for tumorigenesis (Supplementary Table S3). The tumor incidence of LM3/miR-452 group was also significantly higher (Supplementary Table S3). Meanwhile, miR-452 overexpression prompted not only primary (1^°^) but also secondary (2^°^) LM3/miR-452 HCC growth (Figure 2E). Similar results were observed in Huh7 cells (Supplementary Figure S2F, Supplementary Table S3). Finally, *in vivo* metastasis assays showed that the average number of metastatic nodules in liver was dramatically increased about 50% in LM3/miR-452 group (Figure 2F). Taken together, these findings from *in vitro* and *in vivo* assays demonstrated that miR-452 significantly promoted stem-like characteristics of HCC cells.

### MiR-452 knockdown reduced stem-like characteristics of HCC cells

**Figure 3:**
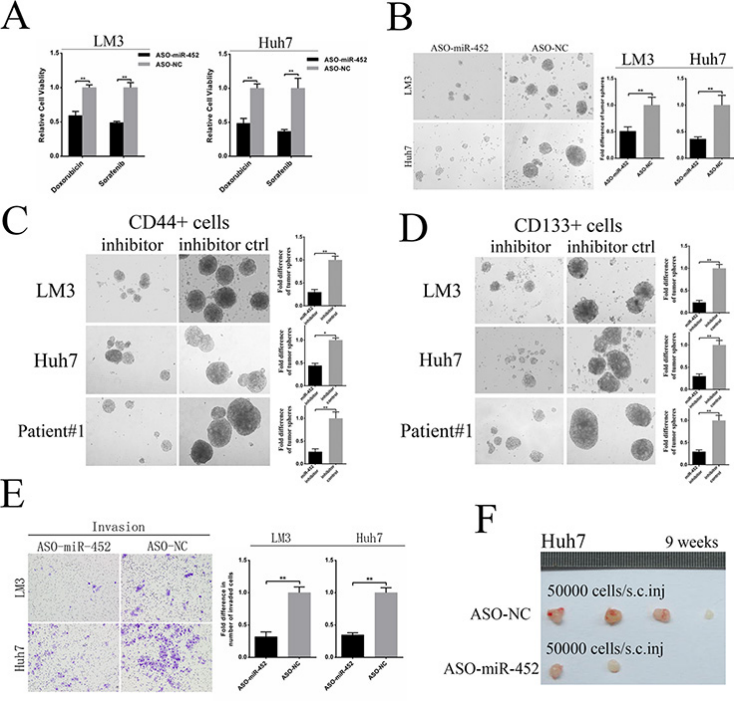
Down-regulation of miR-452 in HCC (**A**) MiR-452 knockdown evidently sensitized LM3 and Huh7 to the chemotherapeutics (**P < 0.001, respectively, *t* test). (**B**) Down-regulation of miR-452 markedly decreased self-renewal ability of HCC cells in the tumor sphere assay. The self-renewal capability of (**C**) CD44+ and (**D**) CD133+ cells sorted from LM3, Huh7 and patient#1 were significantly attenuated by miR-452 inhibitor. (**E**) Invasion efficiencies of LM3 and Huh7 decreased upon miR-452 knockdown. (**F**) The capability of tumorigenicity significantly weakened in the xenograft model of Huh7/ASO-miR-452 cells in comparison with its control.

Further, we also established two miR-452 knockdown stable cell lines (HCC-LM3/ASO-miR-452, Huh7/ASO-miR-452) and their corresponding negative controls (HCC-LM3/ASO-NC, Huh7/ASO-NC) and validated the efficiency (Supplementary Figure S3A). First, we found that miR-452 knockdown decreased the expression of stemness-related gene profile (Supplementary Table S4). Then, miR-452 knockdown cells were much more sensitive to either doxorubicin or sorafenib (Figure 3A). Moreover, miR-452 down-regulation weakened the self-renewal ability of HCC cells presented with smaller size and less hepatospheres (Figure 3B). We also identified that miR-452 inhibitor significantly attenuated the self-renewal capability of CD44+ and CD133+ cells sorted from LM3, Huh7 and patient#1 (Figure 3C, 3D). The migration and invasion efficiencies were also inhibited after miR-452 knockdown (Supplementary Figure S3B, Figure 3E). Eventually, the tumor incidence was also significantly lower upon miR-452 inhibition (Figure 3F, Supplementary Table S5).

### Sox7 was a direct and functional target of miR-452 in HCC

**Figure 4:**
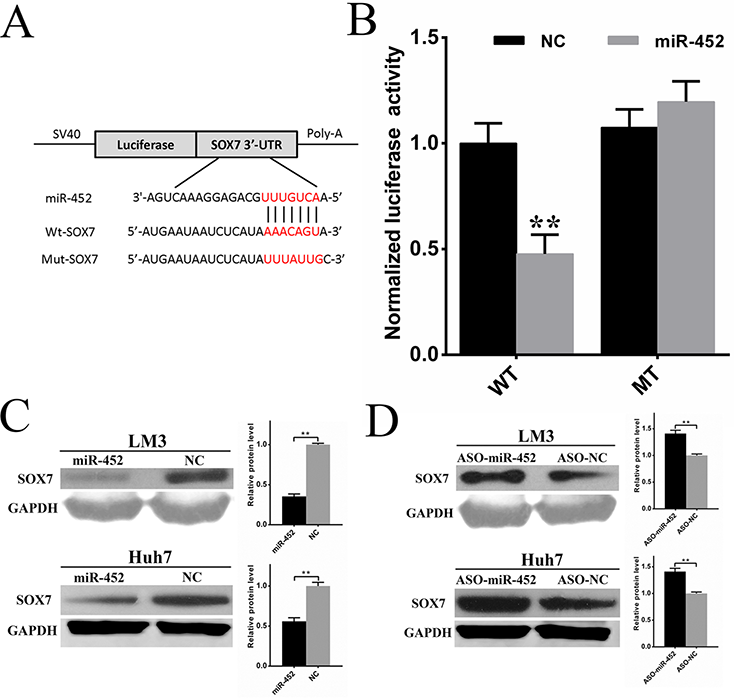
Sox7 was a direct and functional target of miR-452 in HCC (**A**) The sequence of predicted miR-452 binding site and the wild-type (wt-*Sox7*) or mutant (mut-*Sox7*) putative target site in the 3′-UTR of *Sox7* mRNA sequence. (**B**) The luciferase reporter assays were performed in HEK293T cells. The firefly luciferase activity of each sample was normalized to the renilla luciferase activity after 48 h transfection (**P < 0.001, *t* test). The expressions of SOX7 were drastically (**C**) decreased upon miR-452 overexpressed, and (**D**) increased after miR-452 knockdown in both LM3 and Huh7.

To further explore the mechanism by which miR-452 exerted its function, more than 300 genes were predicted to be potential targets using publicly available algorithms (TargetScan, miRanda, PicTar). Considering the reported functions of these genes, several potential genes were further selected. Then, the mRNA expression of these genes were detected by qPCR after transfection of miR-452 mimics or inhibitor into HCC cell lines (data not shown). Finally, we proposed *Sox7* as the candidate gene in our further study. The sequence of the predicted miR-452 binding site and the wild-type (wt-*Sox7*) or mutant (mut-*Sox7*) putative target site in the 3′UTR of *Sox7* mRNA sequence were shown in Figure 4A. The dual-luciferase reporter assays in HEK293T cells revealed that miR-452 overexpression significantly attenuated the activities of luciferase reporters linked to the 3′UTR of *Sox7*, but failed to reduce the luciferase activities of the mutated 3′UTR (Figure 4B). These findings indicated that miR-452 might suppress SOX7 expression through the miR-452 binding sequence in 3′UTR of *Sox7*. Further, the expressions of SOX7 were drastically decreased in both LM3/miR-452 and Huh7/miR-452 cells (Figure 4C), while the expressions of SOX7 were reversed in LM3/ASO-miR-452 and Huh7/ASO-miR-452 cells (Figure 4D). Then, we found that SOX7 was significantly decreased in HCC tissues by immunohistochemistry in training cohort (Supplementary Figure S4A). Notably, miR-452 levels in training cohort were inversely correlated with the expressions of SOX7. The representative immunostaining results of SOX7 were presented in Supplementary Figure S4B, which evidently showed the inverse correlation with miR-452 expression in the same patient (Supplementary Figure S1D). The expression of SOX7 was significantly correlated reciprocally with miR-452 expression (Supplementary Table S1). Kaplan Meier curves identified that decreased SOX7 expression predicted poor overall survival in training cohort (*p* < 0.001; Supplementary Figure S4C).

### Restoration of SOX7 expression inhibited miR-452 correlated stem-like characteristics of HCC cells

**Figure 5:**
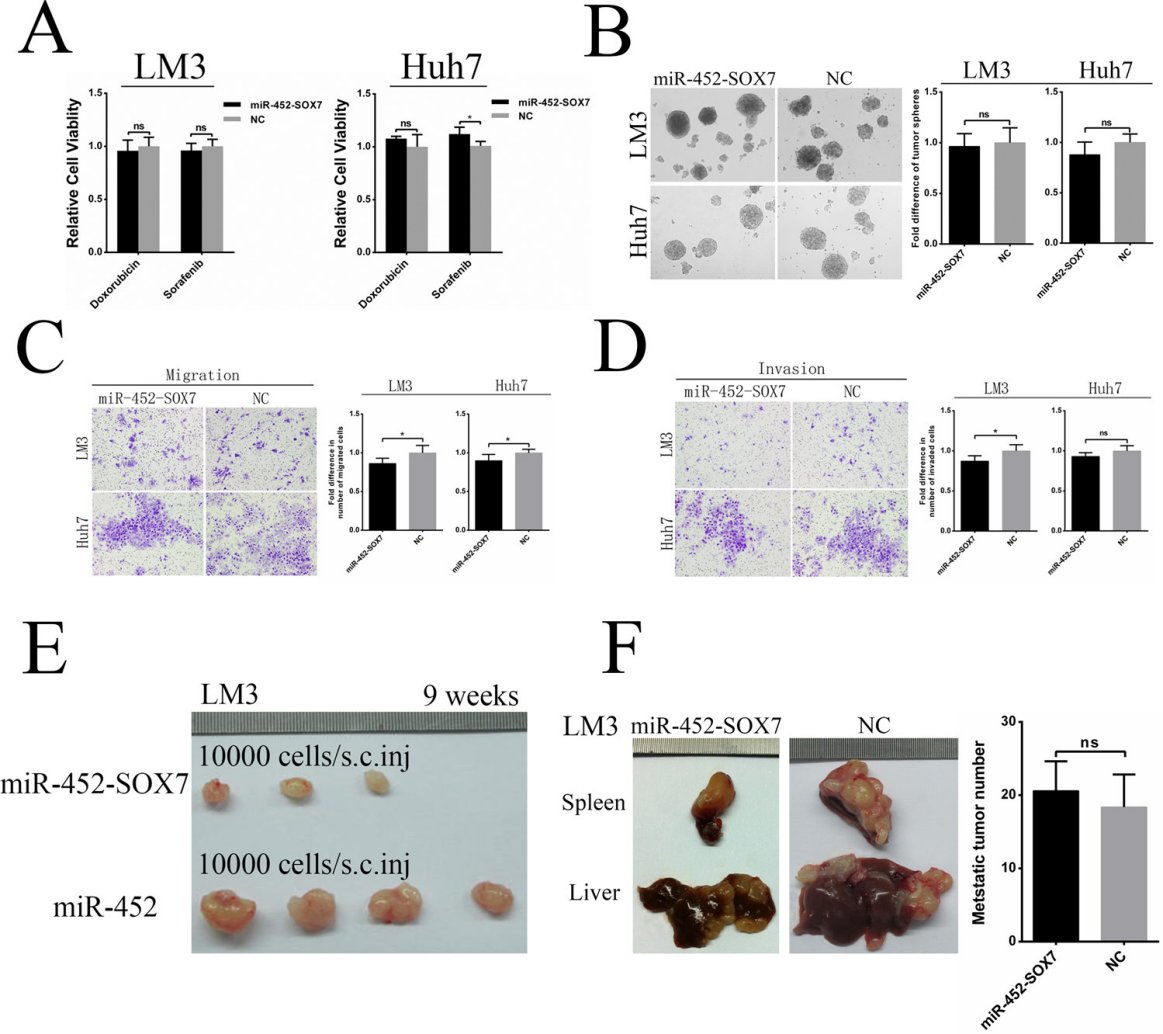
Restoration of SOX7 inhibited miR-452 mediated stem-like characteristics of HCC Re-expression of SOX7 completely or partially, but significantly, eliminated the effects of miR-452 in capabilities of (**A**) Chemoresistance, (**B**) Self-renewal, (**C**) Migration and (**D**) Invasion *in vitro*. Meanwhile, the restoration of SOX7 could also markedly erase the miR-452-mediated competence of (**E**) tumorigenicity in the xenograft models and (**F**) metastasis formation *in vivo* metastasis models.

First, we restored SOX7 expression in miR-452 overexpressed clones of LM3 and Huh7 cells by transfection with lenti-SOX7-ORF, termed LM3/miR-452-SOX7 and Huh7/miR-452-SOX7 (Supplementary Figure S5A, S5B). Then, we investigated whether the effects of miR-452 could be completely or partially eliminated. LM3/miR-452-SOX7 and Huh7/miR-452-SOX7 HCC cells showed a decreased chemo-resistance to doxorubicin and sorafinib (Figure 5A). Moreover, smaller and less hepatospheres were observed upon SOX7 expression restored (Figure 5B). The restoration of SOX7 significantly inhibited migratory and invasive abilities over those of miR-452 overexpressed cells (Figure 5C, 5D). The tumorigenic ability of LM3/miR-452-SOX7 cells was obviously decreased in accordance with the lower tumor incidence (Supplementary Table S6) and smaller tumor size (Figure 5E). Finally, the restoration of SOX7 also drastically inhibited the ability of metastasis formation (Figure 5F) and the number of intrahepatic metastatic nodules showed no significance in comparison with its NC group (Figure 5F). Definitely, these aforementioned results confirmed that miR-452 promoted tumor initiation, stemness and metastasis by targeting SOX7 expression in HCC.

### MiR-452 targeted Sox7 by involving Wnt/β-catenin signaling pathway

**Figure 6:**
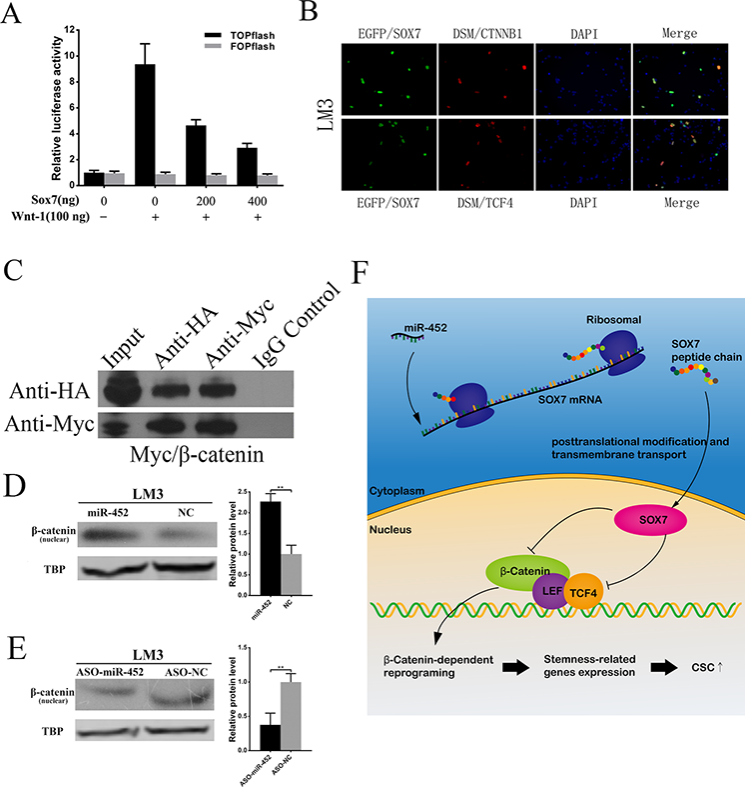
miR-452 targeted SOX7 involving Wnt/β-catenin signaling pathway (**A**) β-catenin/TCF/LEF activity was detected by TOPFLASH reporter assay. FOPFLASH luciferase activity was used as an internal control. (**B**) SOX7 co-localized with CTNNB1 or TCF4 in the nucleus of LM3 by immunofluorescent analysis. (**C**) Co-immunoprecipitation assay showed that SOX7 could interact with β-catenin. IgG was used as a control antibody. Input, total lysate control. (**D**) Upon miR-452 up-regulation, nuclear β-catenin protein increased in LM3. (**E**) While miR-452 down-regulated, nuclear β-catenin decreased. (**F**) Cartoon diagram for the mechanism of miR-452 in the regulation of CSCs by inhibiting Sox7 through activating Wnt/β-Catenin Signaling Pathway.

In our study, we found that the mRNA of β-catenin was significantly elevated when miR-452 was overexpressed (Supplementary Table S2), and markedly declined when miR-452 was down-regulated (Supplementary Table S4). Through literature review, Sox7 was also reported to be able to suppress Wnt/β-catenin activity. Then, we conducted TOPFlash and FOPFlash reporter assay in HEK293T cells. A 9.3 fold increase in β-catenin/TCF/LEF transcriptional activities was presented resulting from the transfection of Wnt1 plasmid. Meanwhile, wild-type SOX7 suppressed β-catenin/TCF/LEF transcriptional activities in a dose-dependent manner in the presence of Wnt1 expression (Figure 6A). Thus, SOX7 was identified to be able to negatively regulate Wnt/β-catenin signaling activity. Further, we wondered that whether SOX7 could directly interact with β-catenin/TCF/LEF transcriptional factor complex. We observed the co-localization of EGFP/SOX7 and DSM/CTNNB1 or DSM/TCF4 mainly in the nucleus of LM3 cells by immunofluorescent microscope (Figure 6B). Consistently, similar observations were presented in Huh7 cells (Supplementary Figure S6A). Then, the HA/SOX7 was proved to be bound with HA/β-catenin (Figure 6C) as well as Myc/TCF4 (Supplementary Figure S6B) by co-immunoprecipitation assay. Next, the nuclear β-catenin was obviously elevated when miR-452 was overexpressed (Figure 6D, Supplementary Figure S6C). While the nuclear β-catenin came to the opposite, when miR-452 was inhibited (Figure 6E, Supplementary Figure S6D). Moreover, the expression of nuclear β-catenin was subverted after the restoration of SOX7 in LM3/miR-452 and Huh7/miR-452 cells (Supplementary Figure S6E, S6F). Altogether, miR-452 was identified to be targeted Sox7 gene activating Wnt/β-catenin signaling pathway leading to the enhancement of stem-like traits of HCC (Figure 6F).

### Combination of doxorubicin and ATRA chemotherapy could efficiently suppress metastasis of miR-452 overexpressed HCC

**Figure 7:**
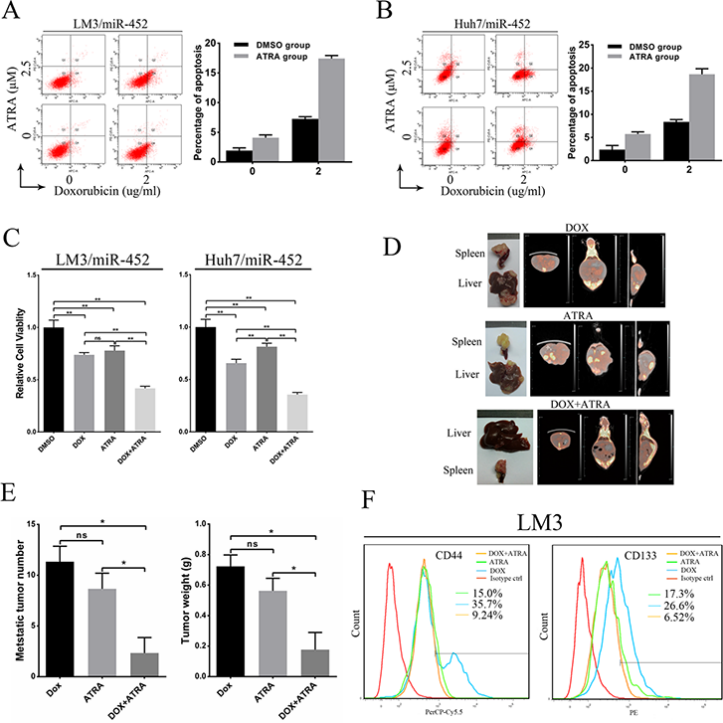
Combination of doxorubicin and ATRA chemotherapy efficiently suppressed metastasis of miR-452 overexpressed HCC Apoptosis of (**A**) LM3/miR-452 and (**B**) HuH7/miR-452 cells receiving the indicated treatments was assessed by Annexin V-APC staining. (**C**) The cell viability was detected after treatment in different groups by CCK8. (**D**) The treatment of *in vivo* metastasis model with doxorubicin and/or ATRA were performed using LM3/miR-452 cells (*n =* 5). (**E**) Further, the average number and total weight of intrahepatic metastatic nodules were showed. (**F**) The percentage of CD44+ and CD133+ subpopulation CSCs were detected derived from those metastatic nodules by FACS.

In our study, we also explored the combined treatment of doxorubicin and ATRA in miR-452 overexpressed HCC. First, the percentage of apoptotic cells were assessed after HCC cells treated by different strategies. The results showed that the proportion of apoptotic cells was lowest in the DMSO control group, and the most amount of apoptosis appeared in the doxorubicin and ATRA combined group (Figure 7A, 7B). The cell viability was also detected after treatment in different groups by CCK8. As shown in Figure 7C, the combined treatment of doxorubicin and ATRA was the most effective. *In vivo* metastasis assay showed that the combination of doxorubicin and ATRA could significantly inhibit miR-452 overexpressed LM3 metastasis. The typical graph of harvested liver and spleen were presented in Figure 7D. In addition, the metastasis formation was monitored by Micro-CT/PET (Figure 7D). The average number and weight of intrahepatic metastatic nodules were significantly decreased in the combined group (Figure 7E). Further, these metastatic nodules were dissociated into single cell suspension and then the percentage of CD44+ and CD133+ subpopulation CSCs were detected by FACS. The percentage of CD44+ cells in the combined doxorubicin and ATRA group (9.24%) was significantly lower than either ATRA group (15%) or doxorubicin group (35.7%) (Figure 7F). Similarly, the percentage of CD133+ cells in the combined doxorubicin and ATRA group (6.52%) was dramatically suppressed compared with either ATRA group (17.3%) or doxorubicin group (26.6%) (Figure 7F). Evidently, the combination of doxorubicin and ATRA treatment exhibited an efficient synergistic effect in suppressing HCC.

## DISCUSSION

In our study, we revealed these following novel findings: (i) Serial passages of the spheres with doxorubicin and sorafenib could enrich CSC populations *in vitro*; (ii) miR-452 was identified to promote stem-like properties of HCC; (iii) Sox7 was a direct and functionally relevant target of miR-452 in HCC; (iv) Combination of doxorubicin and ATRA efficiently suppressed metastasis formation of miR-452 overexpressed HCC.

Serial passages of hepatospheres combined with anti-HCC chemotherapeutics was termed as a new strategy to enrich CSCs [25, 26]. Here, we first reported using doxorubicin and sorafenib to enrich CSCs with the up-regulation of stemness-related genes, an increase of well-known CSC markers, an enhanced self-renewal capability and tumorigenicity.

In our study, we found that miR-452 was up-regulated and predicted poor patient survival and advanced TNM stage in HCC patients. We also identified that miR-452 promoted HCC with higher capacities in chemo-resistance, self-renewal, tumorigenicity and metastasis *in vitro* and *in vivo*, which was consistent with the study of Zheng Q et al [14]. However, in gliomas miR-452 was down-regulated and resulted in the promotion of stem-like characteristics and tumorigenesis [12], which was opposite to these results in HCC. As we all known that cellular transcriptional profiling was changed by spatial and temporal variations in tumor microenvironment [27, 28]. Accumulating evidences demonstrated that an specific miRNA had diverse roles in different microenvironment niches [29]. After all, the potential mechanisms for the differences of miR-452 in regulating CSCs in different malignancies needs to be further studied.

Wnt/β-catenin signaling pathway is an classical one in the regulation of CSCs [21–23]. Moreover, Sox7 blocked tumorigenesis through Wnt/β-catenin signaling pathway [19, 30, 31]. In our study, Sox7 was confirmed to be a direct target of miR-452 and its low expression indicated poor survival of HCC patients. More, miR-452 was identified to promote CSCs by inhibiting *Sox7* through activating Wnt/β-Catenin Signaling Pathway (Figure 6F).

ATRA was identified to promote the differentiation of naive tumor cells in leukemia [24]. However, its therapeutical effect in solid tumors was still undefined, especially for HCC [32]. Zhang et al [33] reported that ATRA could effectively enhance the sensitivity of liver cancer cells to the conventional chemotherapeutic drug cisplatin, which was attributed to ATRA-induced differentiation of HCC CSCs. In our study, we preliminarily demonstrated that the combination of doxorubicin and ATRA could significantly promote HCC CSCs differentiation as well as apoptosis. Notably, the combination treatment could efficiently inhibit tumor metastasis, which might be owed to ATRA-induced differentiation of HCC CSCs followed by the treatment of conventional chemotherapeutics. However, the combined therapy, which targeted both the CSCs subpopulation and the bulk of the tumor cells, might also have some other potential mechanisms in killing HCC tumor cells and more further studies were needed.

Taken together, our study demonstrated that miR-452 promoted stem-like traits of HCC, which might be a potential therapeutic target for HCC. The combination of doxorubicin and ATRA might be an effective and promising strategy in HCC patients.

## MATERIALS AND METHODS

### Clinical specimens and cell lines

The present study enrolled 180 HCC patients who underwent hepatectomy in our center from 2006 to 2014. Human HCC tissues and adjacent normal tissues were immediately snap-frozen in liquid nitrogen before storage at −80°C soon after surgical resection. This study was approved by the Ethical Review Committee of the First Affiliated Hospital, School of Medicine, Zhejiang University, and informed consent was obtained according to the Declaration of Helsinki.

All cells including HepG2, HCC-LM3, Huh7, HEK293T Cells were obtained from Cell Bank of Chinese Academy of Sciences (Shanghai, China) and cultured in Dulbecco's modified Eagle's medium (DMEM; Gibco, USA), supplemented with 10% heat-inactivated FBS (Sigma, USA) and 100 U/mL penicillin/streptomycin. All cells were identified by short tandem repeat (STR) analysis and mycoplasma detection in China center for type culture collection (Wuhan, China).

### Lentiviral-based transfection into HCC cells

For miR-452 up-regulation and down-regulation, LM3 and Huh-7 cells were labeled with luciferase using a lentiviral-based approach. The miR-452 sequence was amplified and inserted into GV254 vector. MiR-452 antisense oligonucleotides (ASO-miR-452) was inserted into GV280 vector. Its negative control (NC) and antisense oligonucleotides negative control (ASO-NC) were used as their corresponding control, respectively. For the restoration of SOX7 expression, Lenti-SOX7-ORF and its control were used to transfect LM3/miR-452 and Huh7/miR-452 cells. Transduced cells were selected with 2 μg/mL puromycin for 14 days.

### Xenograft tumor processing and purification

6-week NOD/SCID mice were purchased from Vital River Laboratories (Beijing, China). The 1^°^ tumor transplantation experiments were performed by subcutaneously injecting HepG2, LM3 or Huh7 cells in 50 ul serum-free medium-Matrigel mixture (1:1). For 2^°^ LM3 or Huh7 tumor transplantation, 1^°^ tumors were dissociated into single cells with Type IV Collagenase (Sigma, USA), Xenografts HCC cells were purified by removing murine cells with a Depletion Kit (Miltenyi Biotech, Germany) before 2^°^ tumor transplantation.

### *In vivo* metastasis assays and chemotherapy treatment

For *in vivo* metastasis assays, 2 × 10^5^ stable transfected LM3 cells in 40 ul serum-free medium were injected into the upper pole of spleen with a microsyringe under anesthesia. After 7 weeks, mice were sacrificed, and their spleens and livers were harvested.

For the treatment of metastasis model, the chemotherapy started immediately after injection. The doxorubicin dose per injection was equivalent to 1.0 mg/kg body weight (i.v. once a week, 6 weeks) and the ATRA dose per injection was equivalent to 3.0 mg/kg body weight (i.p. once a week, 6 weeks). Metastasis formation was finally monitored using Siemens Inveon Micro-CT/PET after 14 weeks. All studies were conducted under the American Association for the Accreditation of Laboratory Animal Care guidelines for humane treatment of animals.

### Human HCC tissue collection and processing

Fresh HCC tissue were collected from one patient (age 60) who underwent surgical hepatectomy in our center and immediately minced into 1 mm^3^ cubes and incubated with Type IV Collagenase for 20 minutes at 37°C. A single-cell suspension was obtained by filtering the supernatant through a 100-μm cell strainer. The removal of CD45+ cells was done with a CD45 deletion kit (Miltenyi Biotech, Germany). Then, isolation of CD44+ and CD44− populations, CD133+ and CD133− populations via MACS were conducted following the manufacture's instruction.

### Plasmids, oligonucleotides and reagents

The HA/SOX7, GFP/SOX7, DSM/CTNNB1 and DSM/TCF4 expressing plasmid were purchased from Invitrogen (USA). The Myc/CTNNB1, Myc/TCF4, Wnt1 expressing plasmid were purchased from Origene (USA). The TCF Reporter Plasmid Kit were brought from Millipore (USA). MiR-452 mimic, miR-452 inhibitor, mimic control and inhibitor control were from Qiagen (Germany). Doxorubicin and Sorafenib were purchased from Santa Cruz (USA). ATRA was obtained from Sigma (USA). Lipofectamine2000 was used for cell transfection according to the manufacturer's instructions.

### *In situ* hybridization

*In situ* hybridization was performed following the protocol developed by Exiqon, Denmark [34]. 5′-DIG and 3′-DIG labeled locked nucleic acid (LNA) modified probes for endogenous miR-452 (Exiqon, Denmark), positive control (U6) and negative control (scramble-miR) were used. Some adjustments were done to get a specific and sensitive detection of miRNA in our sections from FFPE TMA blocks. Tissue staining of miR-452 was scored independently by two pathologists blinded to the clinical data, by multiplication of immunostaining intensity (0–2), and the percentage of immunoreactive cells (0%–100%) resulted in an score ranging from 0 to 2 for each tumor or non-tumor specimens. These patients were classified into high or low expression group according to the mean value of miR-452 expression score.

### Immunohistochemistry

For the construction of TMAs, duplicate 1.0–mm diameter cores of tissue from each sample with each 4 μm sections were punched from paraffin tumor blocks and corresponding non-tumor tissues. Immunohistochemical analysis was performed as previously reported [35]. The primary antibodies against SOX7 was purchased from Novus (1:500, USA). The expression of SOX7 was scored in the same way as mentioned in the miR-452 *in situ* hybridization.

### Sphere formation assay

A total of 1000 single HCC cells were plated onto 6-well Ultra Low Attachment Plates (Corning, USA). Cells were grown in DMEM/F12 (Invitrogen, USA) for 14 days supplemented with B27 (Invitrogen, USA), 20 ng/mL EGF (Peprotech, USA), and 10 ng/mL bFGF (Peprotech, USA), 4 μg/mL insulin (Sigma, USA).

### Luciferase reporter assay

HEK293T cells seeded in 24-well plates were co-transfected with 50 nM of either miR-452 mimic or NC and 100 ng either wild-type or mutant-type 3′UTR of *Sox7* firefly luciferase reporter plasmid. After incubation for 48 h, firefly and renilla luciferase activities were measured by Dual-Luciferase Reporter Assay System (Promega, USA).

### TOP/FOP Flash Wnt reporter assay

HEK293T cells were seeded in 24-well plates and transiently transfected with various amounts (0, 200 and 400 ng) of HA/SOX7, 100 ng Wnt1, TOPFlash or FOPFlash luciferase reporter plasmid (Millipore, Germany). The luciferase activity was measured using the Dual-luciferase Reporter Assay System (Promega, USA).

### Immunofluorescent analysis

For immunofluorescent analysis, LM3 and Huh7 cells were cultured on 6 well plates and transiently co-transfected with either EGFP/SOX7 and DSM/CTNNB1 and DSM/TCF4 expressing plasmids. After 48 hours, the transfected cells were fixed with 4% paraformaldehyde, treated with 0.1% Triton, followed by VECTASHIELD Mounting Medium with DAPI (Vector labs, USA), the fluorescent signals were examined and photographed by fluorescent microscope.

### Co-Immunoprecipitation

For co-immunoprecipitation assay, HEK293 cells were transiently co-transfected with HA/SOX7 and Myc/β-catenin, or Myc/TCF4 plasmids. Dynabeads co-immunoprecipitation Kit (Invitrogen, USA) was used and the process was according to the manufacture's instruction. Western blot was performed to analyze the proteins in the cell lysate and elute [35].

### Quantitative PCR analysis

For mRNA analysis, the cDNA was quantified by qPCR using SYBR Premix Ex Taq (Takara, Japan). For miRNA analysis, it was quantified by qPCR using miScript SYBR Green PCR Kit (Qiagen, Germany). QPCR was performed on a 7500 Fast Real-Time PCR system (Applied Biosystems, USA). RNU6B and GAPDH were used as internal controls. The stemness-related gene profiling primers were in Supplementary Table S7.

### Western blot analysis

Proteins were extracted from cell lines. The nuclear protein extraction was performed by nucleoprotein Extraction Kit (Sangon Biotech, China). Western blot was performed as previously reported [35]. After blocking 2 h with 5% nonfat milk, the membranes were incubated overnight with primary antibodies against SOX7 (Abcam, UK), TCF4 (Cell Signaling, USA), β-catenin (R & D, USA), TBP (Cell Signaling, USA) and GAPDH (Sigma, USA), HA (Abcam, UK), Myc (Abcam, UK).

### Flow cytometric analysis

The antibodies used included PerCP-Cy5.5-conjugated CD44 (eBioscience, USA), PE-Cy7-conjugated EpCAM (eBioscience, USA), APC-conjugated CD90 (eBioscience, USA) and PE-conjugated CD133 (Miltenyi Biotec, Germany). Isotype-matched immunoglobulins were served as controls.

### Cell viability assay

LM3 and Huh7 cells (stably transfected) were cultured in 96-well Plates (Corning, USA) with 5000 single cells per well. After treatment of doxorubicin or sorafenib for 48 h, the relative numbers of viable cells were detected by CCK8 reagents (Dojindo, Japan).

### Migration and invasion assays

Cell migration and invasion assays were performed as previously reported [36]. All assays were performed in triplicates and three fields were counted per filter in each group.

### Annexin V apoptosis assay

Cells in early and late apoptosis were quantified using FACS, following stained with Annexin V and APC.

### Statistical analysis

All experiments were performed in triplicates. All statistical analyses were performed using the statistical software SPSS 20 for Windows (SPSS Inc., Chicago, IL). The significance of various variables for survival was analyzed by univariate and multivariate Cox regression analyses. Survival curves were plotted by the Kaplan–Meier method and compared by the log-rank test. The χ^2^ test or Fisher exact test were used to evaluate the correlation between clinicopathological characteristics and miR-452 or SOX7 expression for HCC patients. The Student *t* test was used for the comparison of measurable variants of two groups. All results were presented as mean ± SD with a P value < 0.05 considered statistically significant, *represented *P* < 0.05, **represented *P* < 0.001.

## SUPPLEMENTARY MATERIALS FIGURES AND TABLES


