# Supplementary material for: MicroRNA-452 promotes stem-like cells of hepatocellular carcinoma by inhibiting Sox7 involving Wnt/β-catenin signaling pathway
Source: Oncotarget. 2016 Apr 5;7(19):28000–12. doi: 10.18632/oncotarget.8584 (PMC5053705; doi:10.18632/oncotarget.8584)
Supplement: Supplementary file 1 [file oncotarget-07-28000-s001.pdf]

MicroRNA-452 promotes stem-like cells of hepatocellular carcinoma by inhibiting Sox7 involving Wnt/β-catenin signaling pathway

Supplementary Materials

Supplementary Table S1: Patient characteristics according to miR-452 expression

Variables	Training cohort			Validation cohort		
	Low (*N* = 45)	High (*N* = 45)	*P* value[a]	Low (*N* = 52)	High (*N* = 38)	*P* value[a]
**Age, years (≤ 55/ > 55)**	23/22	27/18	0.396	27/25	23/15	0.417
**Gender (Female/Male)**	5/40	4/41	1.000	7/45	9/29	0.210
**Maximal tumor size (≤ 5/ > 5)**	21/24	15/30	0.197	29/23	11/27	**0.011**
**Tumor number (< 2/ ≥ 2)**	41/4	38/7	0.522	47/5	36/2	0.694
**Tumor differentiation (Well & moderate/Poor)**	33/12	24/21	**0.049**	42/10	23/15	**0.034**
**PVTT (Yes/No)**	3/42	7/38	0.315	1/51	2/36	0.571
**TNM stage (I/II/III/IV)**	9/14/13/0	2/15/26/2	**0.019**	7/28/14/0	0/17/17/0	**0.023**
**SOX7 expression (low/high)**	18/27	37/8	**< 0.001**			

a, Pearson χ^2-test or Fisher exact test.

Supplementary Table S2: Expression of Stemness-related genes including ABCB1, ABCC1, ABCB5, ABCC2, ABCG2, OCT4, SOX2, NANOG, CTNNB1, TCF4, Notch1, Vimentin in miR-452 overexpressed group and its NC group by qPCR

Stemness-related genes expression of LM3

	**ABCB1**	**ABCC1**	**ABCB5**	**ABCC2**	**ABCG2**	**OCT4**
miR-452/NC	3.026<br>*	4.361<br>**	5.518<br>**	2.549<br>*	5.940<br>**	18.337<br>**
	**SOX2**	**NANOG**	**CTNNB1**	**TCF-4**	**Notch1**	**Vimentin**
miR-452/NC	2.807<br>*	3.315<br>*	9.416<br>**	3.433<br>*	2.292<br>*	4.034<br>*

Stemness-related genes expression of Huh7

	**ABCB1**	**ABCC1**	**ABCB5**	**ABCC2**	**ABCG2**	**OCT4**
miR-452/NC	4.357<br>**	4.429<br>**	25.458<br>**	2.735<br>*	3.231<br>*	5.272<br>**
	**SOX2**	**NANOG**	**CTNNB1**	**TCF-4**	**Notch1**	**Vimentin**
miR-452/NC	8.170<br>*	3.135<br>*	4.670<br>*	3.120<br>*	3.143<br>**	3.433<br>**

(*$P < 0.05$, **$P < 0.001$, *t* test).

Supplementary Table S3: Tumorigenicity of miR-452 group and its NC group HCC cells in NOD/SCID mice

Primary xenograft of LM3 cells (7 weeks)

Cell No.	Tumor/injections			Tumor incidence
	50000	10000	1000	
miR-452	7/8	4/8	1/8	12/24 (50.0%)
NC	2/8	1/8	0/8	3/24 (12.5%)

Secondary xenograft of LM3 cells (7 weeks)

Cell No.	Tumor/injections		Tumor incidence
	50000	10000	
miR-452	6/8	3/8	9/16 (56.25%)

Primary xenograft of Huh7 cells (8 weeks)

Cell No.	Tumor/injections		Tumor incidence
	50000	10000	
miR-452	6/8	4/8	10/16 (62.5%)
NC	1/8	1/8	2/16 (12.5%)

Secondary xenograft of Huh7 cells (7 weeks)

Cell No.	Tumor/ injections		Tumor incidence
	50000	10000	
miR-452	5/8	3/8	8/16 (43.8%)

Supplementary Table S4: Expression of Stemness-related genes including ABCB1, ABCC1, ABCB5, ABCC2, ABCG2, OCT4, SOX2, NANOG, CTNNB1, TCF4, Notch1, Vimentin in ASO-miR-452 group and its ASO-NC group by qPCR

Stemness-related genes expression of LM3

	ABCB1	ABCC1	ABCB5	ABCC2	ABCG2	OCT4
ASO-miR-452/ASO-NC	0.457<br>*	0.420<br>*	1.082<br>n.s.	0.493<br>*	0.139<br>*	0.322<br>*
	**SOX2**	**NANOG**	**CTNNB1**	**TCF-4**	**Notch1**	**Vimentin**
ASO-miR-452/ASO-NC	0.490<br>*	1.147<br>n.s.	0.183<br>*	0.220<br>*	0.384<br>*	0.280<br>*

Stemness-related genes expression of Huh7

	ABCB1	ABCC1	ABCB5	ABCC2	ABCG2	OCT4
ASO-miR-452/ASO-NC	0.168<br>*	0.881<br>n.s.	0.272<br>*	0.252<br>**	0.187<br>*	0.144<br>**
	**SOX2**	**NANOG**	**CTNNB1**	**TCF-4**	**Notch1**	**Vimentin**
ASO-miR-452/ASO-NC	0.886<br>n.s.	0.447<br>n.s.	0.197<br>**	0.462<br>*	0.857<br>n.s.	0.315<br>**

(*$P < 0.05$, **$P < 0.001$, *t* test).

Supplementary Table S5: Tumorigenicity of ASO-miR-452 group and its ASO-NC group HCC cells in NOD/SCID mice

Primary xenograft of Huh7 cells (9 weeks)

Cell No.	Tumor/injections		Tumor incidence
	50000	10000	
ASO-miR-452	2/6	0/6	2/12 (16.67%)
ASO-NC	4/6	1/6	5/12 (41.67%)

Supplementary Table S6: Tumorigenicity of miR-452-SOX7 group and its NC group HCC cells in NOD/SCID mice

Primary xenograft of miR-452-Sox7, miR-452 and NC group in LM3 cells (9 weeks)

Cell No.	Tumor/injections	Tumor incidence
	10000	
MiR-452	4/8	50%
miR-452-Sox7	3/8	37.5%
NC	1/8	12.5%

Supplementary Table S7: Sequences of qPCR primers

Gene	Primer Sequence (Forward, 5′–3′)	Primer Sequence (Forward, 5′–3′)
ABCB1	AAATTGGCTTGACAAGTTGTATATGG	CACCAGCATCATGAGAGGAAGTC
ABCC1	CTCCTCCTATAGTGGGGACATCAG	GTAGTCCCAGTACACGGAAAG
ABCB5	TCTGGCCCCTCAAACCTCACC	TTTCATACCGCCACTGCCAACTC
ABCC2	ATGCAGCCTCCATAACCATGA	CTTCGTCTTCCTTCAGGCTATTCA
ABCG2	ACTCAGTTTATCCGTGGTGTG	CCTGCTTAGACATCCTTTTCA
OCT4	TATTCAGCCAAACGACCATCT	ACGAGGGTTTCTGCTTTGC
SOX2	ATGGGTTCGGTGGTCAAGTC	GCTCTGGTAGTGCTGGGACAT
NANOG	CCGAAGAATAGCAATGGTG	CCTGGTGGTAGGAAGAGTAAA
CTNNB1	ACAACTGTTTTGAAAATCCA	CGAGTCATTGCATACTGTCC
TCF4	GTGGACATTTTACTGGCTCAA	AAAAGGTGGAGAGAGATTGTCA
NOTCH1	CCTGAGGGCTTCAAAGTGTC	CGGAACTTCTTGGTCTCCAG
Vimentin	GAGAACTTTGCCGTTGAAGC	TCCAGCAGCTTCCTGTAGGT
GAPDH	ATGGGGAAGGTGAAGGTCG	GGGGTCATTGATGGCAACAATA

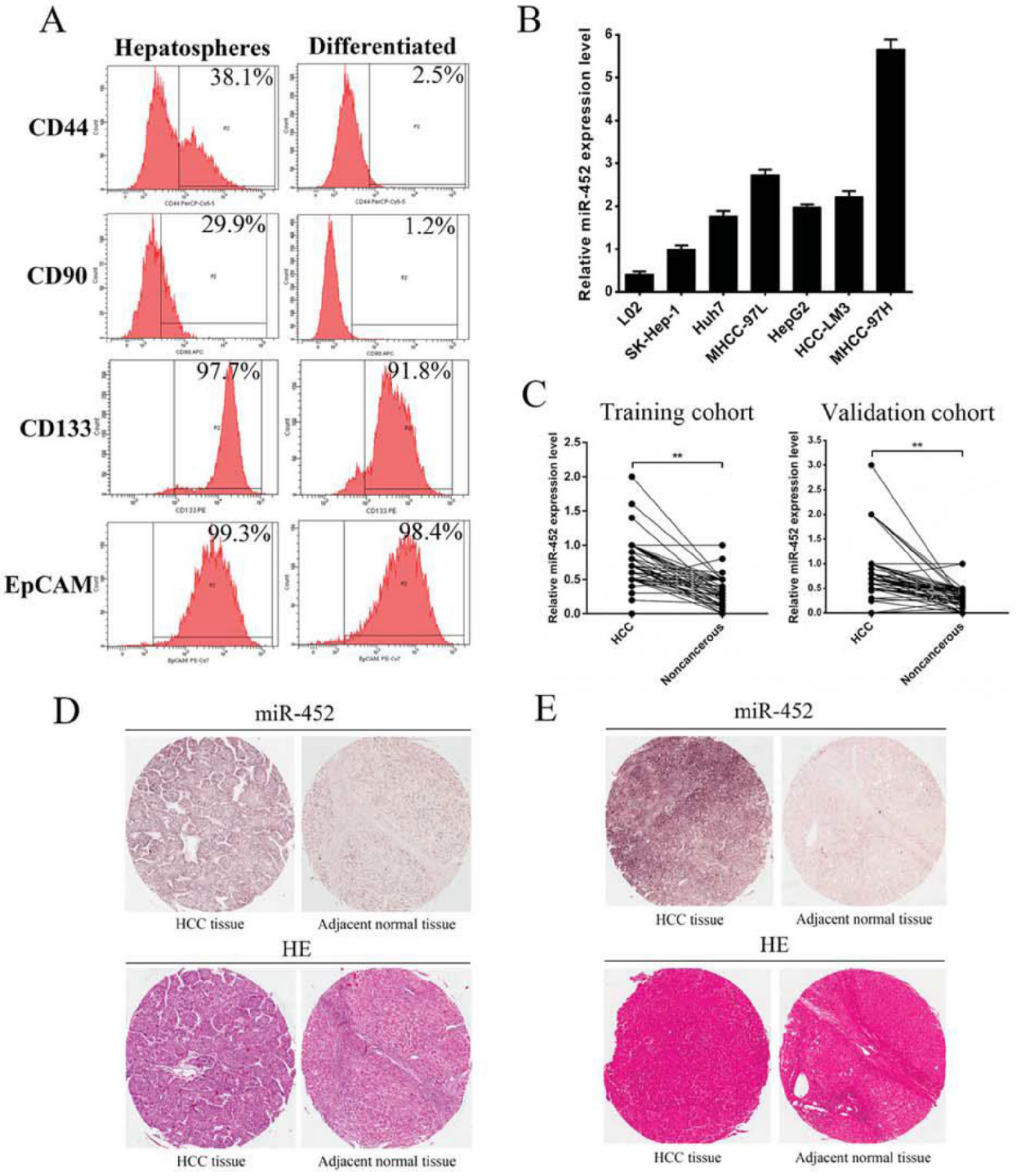

Supplementary Figure S1: MiR-452 indicated poor survival in HCC. (**A**) In the chemo-resistant hepatospheres, liver CSC markers including CD44, CD90, CD133 and EpCAM significantly elevated compared with the differentiated colones. (**B**) The expression of miR-452 in various HCC cell lines significantly higher than L02 cells by qPCR analysis. (**C**) The expression of miR-452 in HCC tissues also markedly elevated than adjacent liver tissues by ISH in the tissue microarray and the quantitative results presented both in the training cohort and validation cohort. Representative images of miR-452 expression by ISH as well as corresponding HE graphs in tissue microarray were showed both for the (**D**) training cohort and (**E**) validation cohort.

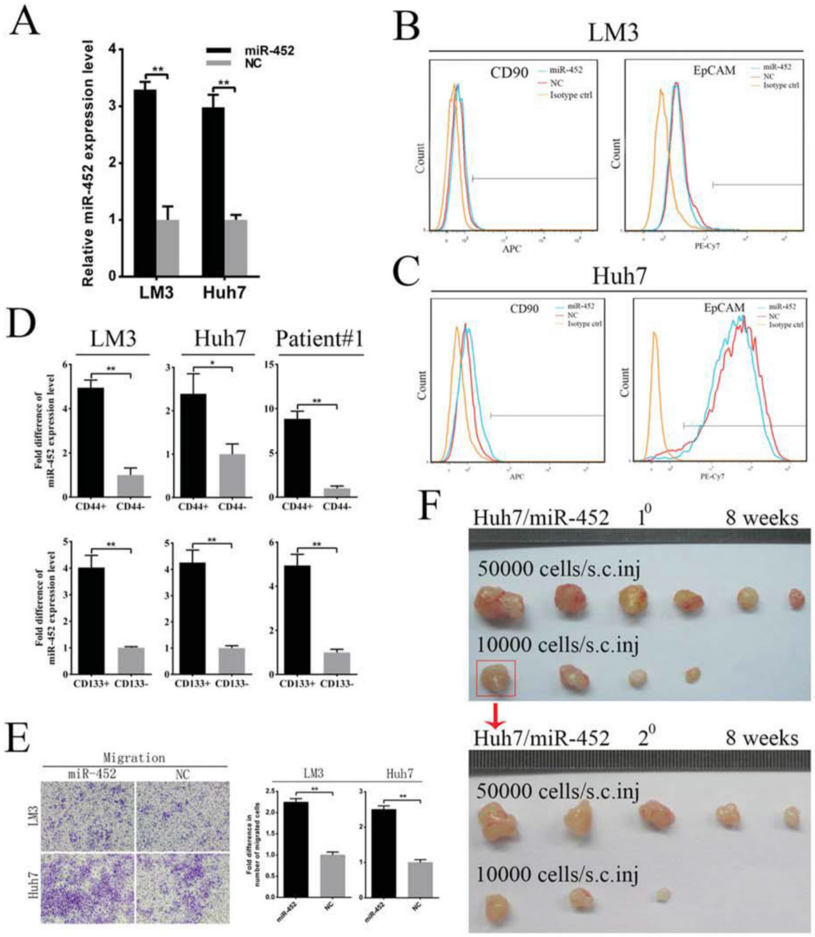

Supplementary Figure S2: Up-regulation of miR-452 in HCC. (**A**) the expression of miR-452 up-regulated about 3 folds compared with negative control in LM3 and Huh7 by qPCR. Representative FACS for CD90 and EpCAM markers in (**B**) LM3 and (**C**) Huh7 after miR-452 overexpressed, which showed no significant change after miR-452 overexpressed compared with corresponding negative control. (**D**) The expression of miR-452 in CD44+ and CD133+ cells sorted from LM3, Huh7 and Patient#1 by MACS were markedly higher than that in CD44- and CD133- HCC cells. (**E**) Cell migration assay demonstrated that miR-452 efficiently promoted HCC migration *in vitro*. (**F**) The capability of tumorigenicity significantly enhanced both in the primary and secondary xenograft model of Huh7/miR-452 cells in comparison with Huh7/NC cells. Representative images were shown for the tumors derived from Huh7/miR-452 cells subcutaneously injected into NOD/SCID mice.

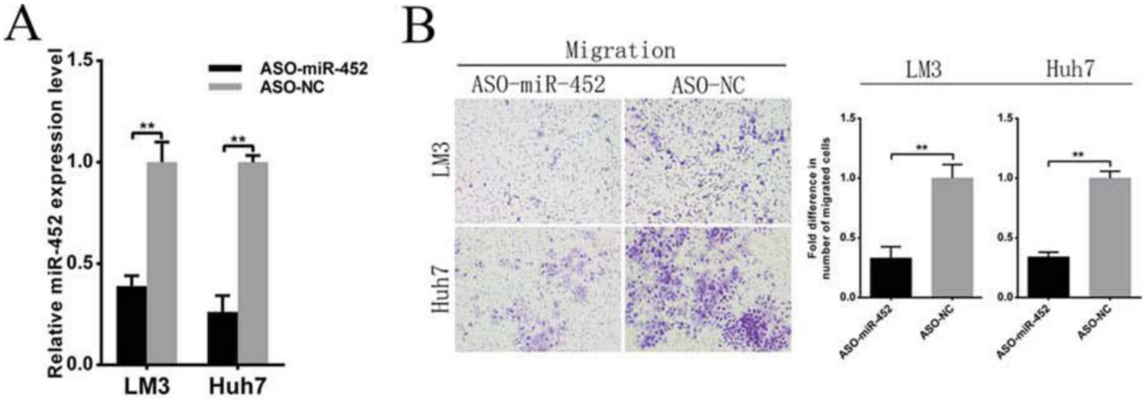

Supplementary Figure S3: Down-regulation of miR-452 in HCC. (**A**) Knockdown of miR-452 in LM3 and Huh-7 cells resulted in more than 60% decrease compared with their corresponding controls. (**B**) Transwell assay indicated that migration efficiencies of LM3 and Huh7 cells decreased upon miR-452 knockdown.

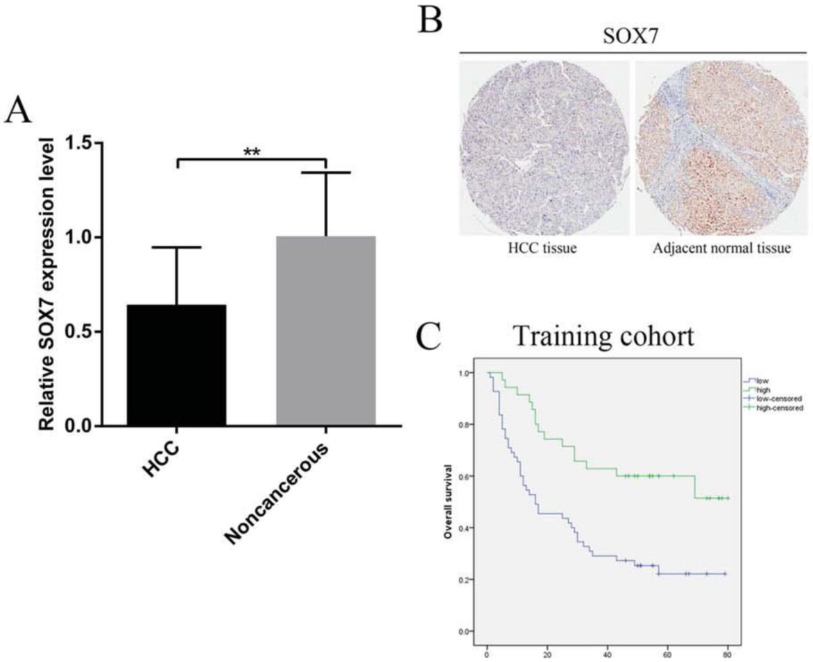

Supplementary Figure S4: MiR-452 targeted *Sox7*. (**A**) The expression of SOX7 protein significantly decreased in HCC tissues compared with adjacent noncancerous tissues by IHC. (**B**) Representative images of SOX7 expression in the training cohort were showed. (**C**) Kaplan Meier analysis of HCC patients with low- versus high-expression of SOX7 in the training cohort ($N = 90$, $P < 0.001$, log-rank). Patients with low expression of SOX7 owned a poor overall survival.

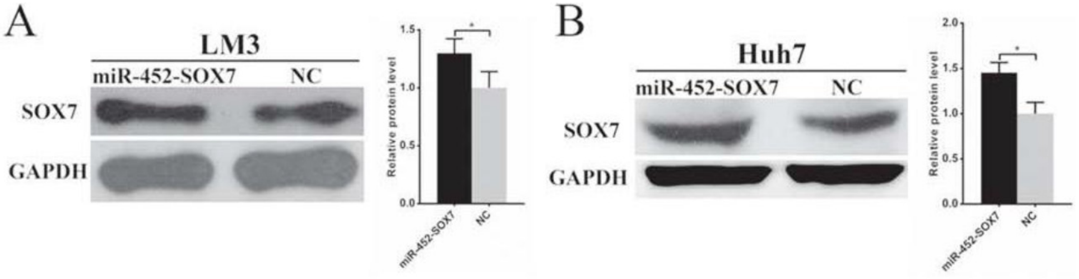

Supplementary Figure S5: The Sox7 ORF was successfully transfected into a miR-452 overexpressed clone of (A) LM3 and (B) Huh-7 cells; the expression level was comparable to the non-target control.

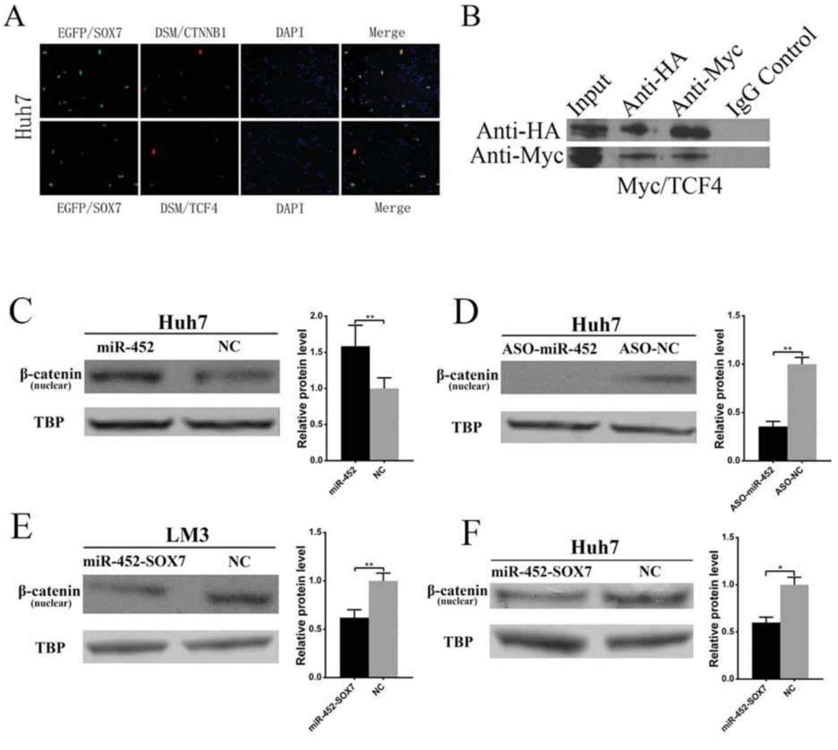

Supplementary Figure S6: miR-452 regulated Sox7 through activation of Wnt/β-catenin signaling pathway. (**A**) Sox7(EGFP/Sox7) co-localized with CTNNB1 (DSM/CTNNB1) or TCF4 (DSM/TCF4) in the nucleus of Huh7 cells by immunofluorescent analysis. (**B**) Co-immunoprecipitation assay showed that Sox7 (HA/Sox7) could interact with TCF4 (HA/Myc). IgG was used as a control antibody. Input, total lysate control. (**C**) Upon miR-452 up-regulation, nuclear β-catenin protein increased in Huh-7. (**D**) While miR-452 down-regulated, nuclear β-catenin decreased. Upon the restoration of SOX7 protein, nuclear β-catenin significantly decreased in both LM3 (**E**) and Huh7 (**F**) cells.
